# Differential causes of masticatory muscle disorders in dogs: a review of diagnosis, treatment and long-term management

**DOI:** 10.3389/fvets.2026.1750095

**Published:** 2026-02-10

**Authors:** Rachelle Fisher, Stephanie Goldschmidt, Marguerite F. Knipe, Maria Soltero-Rivera, Natalia Vapniarsky, Boaz Arzi

**Affiliations:** 1 Nebraska Dentistry and Oral Surgery for Animals, Omaha, NE, United States; 2 Department of Surgical and Radiological Sciences, University of California, Davis, Davis, CA, United States; 3 Department of Pathology, Microbiology and Immunology, School of Veterinary Medicine, University of California, Davis, Davis, CA, United States

**Keywords:** biopsy, canine, computed tomography, difficulty in opening and closing the mouth, pain, trismus

## Abstract

Masticatory muscle disorders in the dog are complex and challenging cases to manage. Patients may present with inability or difficulties in opening or closing the mouth, making dysphagia and respiratory compromise major concerns. Time is of the essence in these circumstances, and prompt and accurate diagnosis and treatment are required to prevent potentially life-threatening complications from occurring. Several local and systemic disease processes may cause masticatory muscle dysfunction further complicating the diagnostic process. Current literature on masticatory muscle disorders is widespread with many recommendations in veterinary medicine extrapolated from human medicine. The goal of this paper is to synthesize the current literature and illuminate areas that require additional research.

## 1 Introduction

The muscles of mastication are responsible for essential motor functions such as chewing and biting. In patients with the inability to open the mouth, breathing may be negatively impacted due to airway patency issues and respiratory distress, especially in dogs with brachycephalic skull configuration (1). The masticatory muscles responsible for closing the mouth and elevating the mandible include the temporalis, masseter, medial pterygoid, and lateral pterygoid. The rostral belly of the digastricus is considered a complementary muscle of mastication and is responsible for opening the mouth. This action is supplemented by the lateral pterygoid, which helps to depress the mandible while opening. All of these muscles are innervated by the mandibular branch of the trigeminal nerve (CN V) with exception of the caudal belly of the digastricus muscle, which is innervated by the facial nerve (CN VII) (2).

Primary dysfunction of the masticatory muscles typically manifests as changes in jaw mobility (1). Trismus is a spasm of the muscles of mastication, resulting in inability to open the mouth (3). Conversely, mandibular paralysis or “dropped jaw” can manifest as an inability to close the mouth (4). Several systemic and localized disease processes may cause dysfunction of the masticatory muscles that present early on as difficulty in opening or closing the mouth with or without obvious pain (Table 1). If patients are not promptly and accurately diagnosed and treated, severe consequences may develop with some causing life-threatening sequelae (1). The goal of this review is to inform on the spectrum of masticatory muscles disorders, synthesize the current literature to assist clinicians in approaching these challenging cases, and highlight knowledge gaps that require further research.

**Table 1:** Differentials for masticatory muscle disorders in the dog.

Pathophysiologic cause	Disorder
Inflammatory	Masticatory Muscle Myositis (MMM)† Polymyositis (PM) Diffuse Extraocular myositis (EOM) Laryngeal Otitis media/interna* Retrobulbar abscesses*
Infectious	Parasitic Neosporosis Toxoplasmosis Hepatozoonosis Trypanosomiasis Trichinosis* Sarcocystosis Bacterial Leptospirosis Clostridial Tetanus Rickettsial *Ehrlichia canis* Fungal* Sporotrichosis* Systemic mycosis*
Endocrine	Hyperadrenocorticism* Hypothyroidism*
Musculoskeletal	Myositis ossificans (MO)† Myositis Ossificans Progressiva (MOP) Myositis Ossificans Traumatica (MOT) Acute compartment syndrome (ACS) Craniomandibular osteopathy (CMO)* Sarcopenia* Systemic lupus erythematous (SLE)* TMJ disorders*† Luxation/subluxation* Fracture* Ankylosis/pseudoankylosis* Osteoarthritis (OA)*
Neoplasia	Trigeminal nerve sheath tumor (TNST) Lymphoma Cancer cachexia
Neoplasia	Trigeminal nerve sheath tumor (TNST) Lymphoma Cancer cachexia
Drug-Induced	Cimetidine* Trimethoprim-sulfadiazine* D-penicillamine*
Trauma	Bite wounds† Motor vehicle accidents* Training injuries†

*Additional differentials listed but not discussed in this review due to lack of published case reports in veterinary literature. †Potential for primary masticatory muscle involvement.

## 2 Inflammatory myopathies

Inflammatory myopathies are defined as those in which skeletal muscles contain infiltrates that are both cellular and nonsuppurative (5). In dogs, focal and generalized inflammatory myopathies have been recognized (5–9), and can be further categorized as immune-mediated, idiopathic, or secondary to infectious disease (9). The most common inflammatory myopathies of dogs that can affect the muscles of mastication are masticatory muscle myositis (MMM), polymyositis (PM), and generalized myositis from infectious disease (5). Extraocular myositis (EOM) and dermatomyositis (DM) are also inflammatory myopathies of dogs, but do not directly cause masticatory muscle dysfunction (6).

A canine Overlap Syndrome (OS) has also been reported in the literature in which dogs display clinical signs consistent with both PM and MMM. OS results in cellular infiltrates being found in limb muscles and masticatory muscles, a characteristic finding of PM, and also have positive 2 M antibodies (5). Additional research is needed to further explore the prevalence, severity, and prognosis of OS inflammatory myopathies.

The diagnostic workup for inflammatory myopathies includes laboratory work (complete blood count/chemistry panel), advanced diagnostic imaging, and muscle biopsy with histopathologic review. Bloodwork changes routinely include elevated creatine kinase (CK) due to myofiber necrosis (5). Severe myofiber necrosis in cases of inflammatory myopathies may also result in elevated levels of alanine aminotransferase (ALT) or aspartate aminotransferase (AST) (9). A neutrophilia, secondary to leukocytosis, may also be present (10).

**Figure 1:**
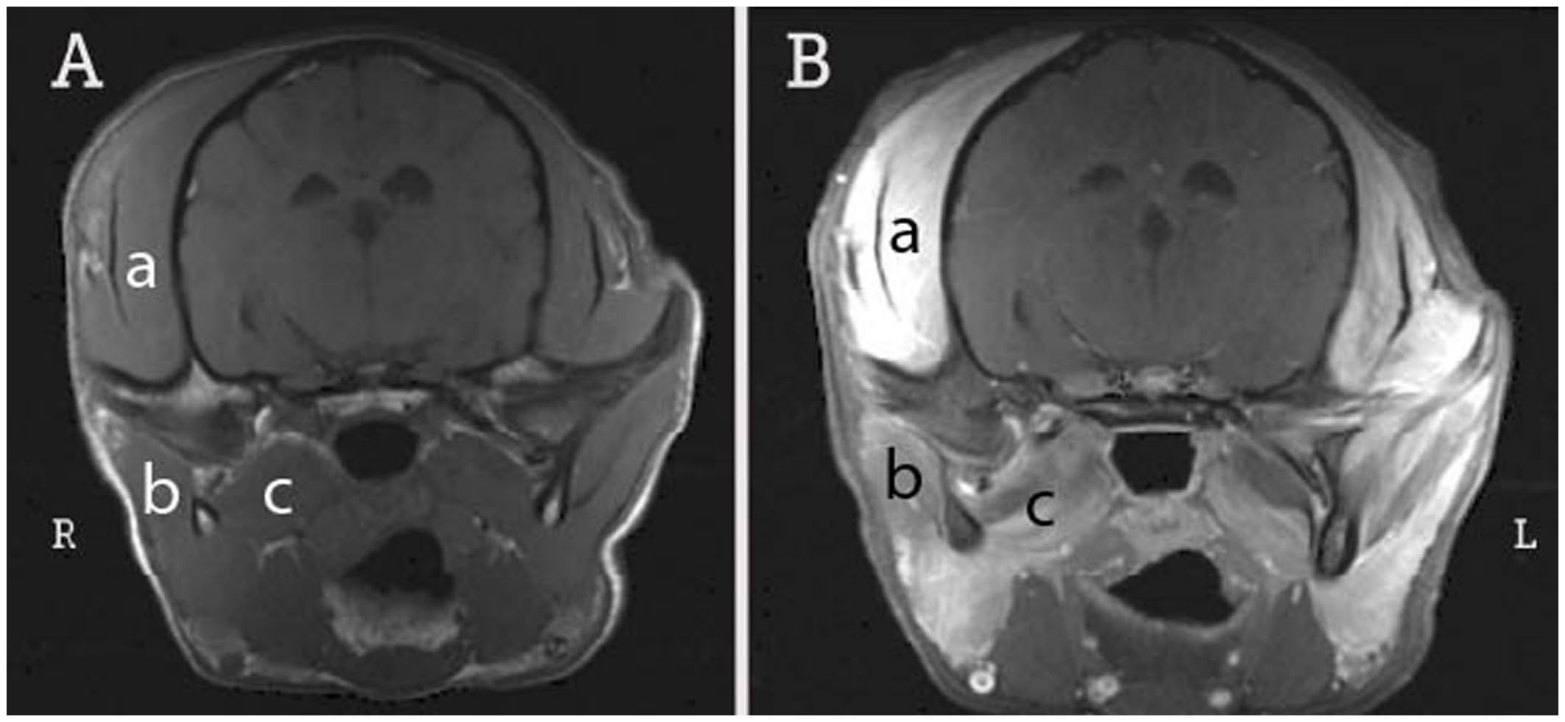
Transverse T1-weighted MR images pre-contrast **(A)** and post-contrast **(B)** at the level of the temporomandibular joint in an 8-month-old FS miniature pinscher dog with immune-mediated masticatory myositis (MMM). There is bilateral contrast enhancement of the temporalis (a), masseter (b), and pterygoid (c) muscles, and asymmetrical temporalis atrophy, worse on the left. The patient’s 2 M antibody titer was positive at 1:500.

Advanced diagnostic imaging in the form of contrast computed tomography (CT) or magnetic resonance imaging (MRI) is prudent to assess the nature and extent of myositis (8–11). In inflammatory myopathy cases, muscles are initially inflamed and edematous, appearing enlarged and hypoattenuating on CT pre-contrast (11). After contrast administration, non-homogenous or heterogenous enhancement is detected due to the increased vascularity of inflammatory change (12). Areas that lack contrast enhancement can be present, which correspond to areas of muscle necrosis (11). MRI is superior in inflammatory myopathy cases when differentiation is needed amongst fat, fibrosis, cellular infiltrates, edema, and calcification (7). On both T1 and T2 weighted images, muscles affected by inflammatory myopathies are poorly marginated and diffuse, appearing hyperintense (8, 9). When contrast is administered, marked enhancement is noted in affected muscles and areas of necrosis do not have enhancement (Figure 1) (8).

**Figure 2:**
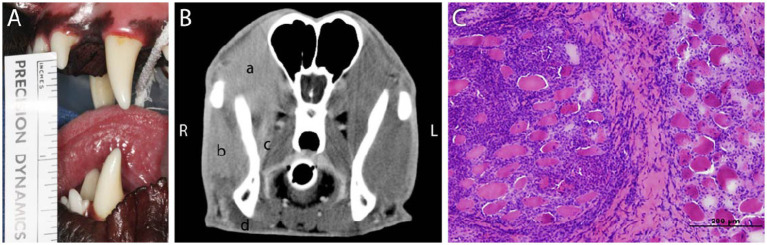
**(A)** A 1-year-old male Rottweiler dog that was presented with gradual inability to open the mouth with an interincisal distance of 38 mm on maximal opening. **(B)** Contrast-enhanced CT image demonstrating contrast enhancement of the right temporalis (a), right masseter (b), and pterygoid muscles (c). Note that no contrast is enhanced in the digastricus muscles (d) as it typically does not contain type 2 M fibers and is not involved in MMM. **(C)** Biopsy and histology of the temporal muscle shows myonecrosis and severe endomyseal mononuclear infiltration, confirming the diagnosis of active masticatory muscle myositis.

Confirmatory diagnosis of an inflammatory myopathy is made through muscle biopsy with histopathologic detection of inflammatory change present (Figure 2) (7). Biopsy sites are determined based on localization of contrast enhanced areas in CT and MRI studies (8, 11).

### 2.1 Masticatory muscle myositis (MMM)

MMM is an immune-mediated inflammatory myopathy that targets the muscles of mastication containing type 2 M myofibers (masseter, temporalis, medial pterygoid, and lateral pterygoid) (12, 13). These muscles are innervated by the mandibular branch of the trigeminal nerve and contain a type 1 myofiber variant along with the targeted type 2 myofibers. Additionally, and uniquely to the muscles of mastication (particularly masseter, temporalis, and pterygoids), there are type 2 M fibers (14). The rostral belly of the digastricus is also innervated by the trigeminal nerve (the caudal belly is innervated by the facial nerve), but it contains only type 1 and 2A myofibers and is not affected in cases of MMM (15).

MMM occurs due to circulating autoantibodies that target type 2 M myofiber antigens, specifically masticatory myosin binding protein-C (mMyBP-C) (14). Once bound, immune complexes are created and incite muscle inflammation, destroying type 2 M myofiber cell membranes (12). This leads to prolonged muscle contraction and the typical MMM clinical signs (16). Currently, the inciting cause of autoantibody formation is not known (14). Theories revolve around the notion of molecular mimicry in which antibodies or T cells are created from exposure to an infectious agent (17). These antibodies or T cells then cross react with self-antigens, similar to the development of rheumatoid arthritis in humans (14). Others propose that the inciting factor may be damage to muscle fibers that release large fragments of myosin, resulting in anti-myosin antibody production (18).

Historically, MMM has been referred to as eosinophilic or atrophic myositis (14). While these terms are not commonly used today, they are helpful descriptors of the two phases of disease. The acute phase of MMM involves gradual inability to open the mouth and eventually trismus, jaw discomfort, and masticatory muscle swelling and pain (13). Ocular changes, such as exophthalmos may also be evident in the acute phase and have been reported in up to 44% of dogs (14). In these cases, it is important to rule out EOM (6). Other clinical signs that have been reported in the acute phase include fever and lymphadenopathy (14). The chronic phase is identified visually as masticatory muscle atrophy with restricted jaw movement from fibrosis (12). Not all cases include a clear acute phase and involve slow and progressive muscle atrophy instead (13). When the classic presentation of MMM is apparent, early detection and treatment in the acute phase are the key to preventing permanent muscle atrophy and loss of jaw mobility (12).

Dogs are typically diagnosed between 1 and 3 years of age, but there have been several reported cases of dogs being diagnosed at less than a year of age (14, 19–21). This average age is based on older retrospective studies and case reports, so updated reports on age of onset and diagnosis are needed (19). Over reported breeds include golden retriever, German shepherd, Labrador retriever, and Doberman pinscher dogs, yet no studies compare the breeds to the hospital distribution of breeds precluding diagnosis of a true breed prevalence (14). Two separate case reports have been documented on multiple Cavalier King Charles spaniels (CKCS) developing an atypical form of the disease that includes ocular abnormalities (20, 21). Additionally, there have been two documented cases of dogs being diagnosed with myasthenia gravis and MMM concurrently, suggesting another form of OS (10, 22).

Diagnostic workup for MMM is consistent with other inflammatory myopathies and includes bloodwork and advanced diagnostic imaging. Common blood work abnormalities include hyperglobulinemia, anemia, and proteinuria (14, 19). CK can be elevated during the acute phase but returns to normal during the chronic phase (7). On CT imaging, MMM appears as hypoattenuation of affected masticatory muscles prior to contrast being given, which is followed by heterogeneous or random contrast enhancement after administration (Figure 2) (11, 12). On MRI, MMM findings are symmetrical, non-homogenous, and hyperintense foci in the affected muscles of mastication (Figure 1). Gradient-echo short T1 inversion recovery (GE STIR) sequences are sensitive to inflammatory processes such as MMM, because they saturate fatty tissue and allow for better muscular structure definition. Fluid buildup and edema early on in the course of MMM cause prolonged T2 relaxation and make affected muscles appear hyperintense on T2 weighted images. No changes are detected on T1 relaxation; therefore, affected muscles appear isointense or marginally hypointense. After contrast administration, T1 weighted images become mildly enhanced in affected muscle bellies (23). MRI signal intensity coming from affected muscles is useful for staging MMM (24).

In addition, a confirmatory blood test for MMM can be performed via the serum 2 M ELISA antibody titer test (12). This test is very specific (100%) and highly sensitive (85–90%) (25). Importantly, a false negative is possible if immunosuppressive doses of corticosteroids at 2 mg/kg/day were administered within seven to ten days of testing or in patients with chronic MMM (26). Patient prognostic information cannot be derived from titer results (25). Positive titers results include 1:500 (low), 1:1000 (medium), or 1:4000 (high) (12, 14). A titer of 1:100 (borderline) can be due to early disease course or errantly decreased from corticosteroid use (12, 25). The canine 2 M ELISA antibody titer test can also be used in feline patients because of the cross-reacting ability of 2 M antibodies (13).

Last, other advanced diagnostics for more challenging cases may include electromyography (EMG) and muscle biopsy with histopathology. EMG in MMM patients reveals spontaneous activity of the muscles of mastication only, differentiating it from polymyositis (14). This shows up as positive sharp waves and fibrillations (23). Patients in the chronic phase of MMM may have normal EMG reports due to the severe atrophy that is present (14).

Muscle biopsy is indicated to confirm the diagnosis, especially when muscle atrophy or fibrosis are present (14). Most commonly used biopsy locations are the temporalis muscle and superficial portions of the masseter muscle (27). MMM cellular infiltrates are different from those found in other inflammatory myopathies (5). These infiltrates are predominately B-cells, macrophages, and dendritic cells (6, 11). In the authors experience, the inflammatory infiltrate can be rich in neutrophils and their presence should not exclude MMM as a differential. Also, histopathologic analysis allows for the amount of fiber lost and severity of fibrosis to be documented, helping determine prognosis and likely response to therapy (14).

The mainstay of MMM treatment is immunosuppression with corticosteroids (Table 2) (28). Prednisone is typically prescribed at 2 mg/kg PO q12h for patients in the acute phase (14). Yet, there is no evidence to support that a dose this high is actually warranted, and additional research and reports on lower corticosteroid dosing in long term management of MMM patients is needed. The authors of this manuscript often treat at 2 mg/kg PO q24h with positive results.

**Table 2:** Pharmacological management for masticatory muscle disorders in the dog.

Drug	Dose	Route
Masticatory muscle myositis (MMM)		
Prednisone	2 mg/kg q12-24 h	PO
Azathioprine (+ Prednisone)	2 mg/kg q24-48 h	PO
Cyclosporine (+ Prednisone)	5 mg/kg q12h	PO
Leflunomide	2–3 mg/kg q24h	PO
Mycophenolate mofetil	10 mg/kg q12h	PO
Oclacitinib	1 mg/kg q12h	PO
Polymyositis (PM)		
Prednisone	1–2 mg/kg q12h	PO
Azathioprine (+ Prednisone)	2 mg/kg q24h	PO
Leflunomide (+ Prednisone)	4 mg/kg q24h	PO
Methotrexate	2.5 mg/m^2^ q24h	PO
Neosporosis		
Clindamycin	12–25 mg/kg q12h for 4 weeks	PO
Trimethoprim sulfadiazine + Pyrimethamine	15–20 mg/kg q12h for 4 weeks + 1 mg/kg q24h for 4 weeks	PO + PO
Toxoplasmosis		
Clindamycin	10 mg/kg q8h	PO
Trimethoprim sulfadiazine + Pyrimethamine	15 mg/kg q12h + 1 mg/kg q24h	PO + PO
Azithromycin	10 mg/kg q24h	PO
Leishmaniasis		
Meglumine antimoniate + Allopurinol	75–100 mg/kg q24h for 4–8 weeks + 10 mg/kg q12h for 6 months	SQ + PO
Amphotericin B	0.1–1 mg/kg q24h or twice weekly for 2 months	IV
Miltefosine + Allopurinol	2 mg/kg q24h + 10 mg/kg q12h for 6 months	PO + PO
Trypanosomiasis		
Amiodarone + Itraconazole	7.5 mg/kg q24h + 10 mg/kg q24h	PO + PO
Sarcocystosis		
Clindamycin	13 mg/kg q12h	PO
Decoquinate	10–20 mg/kg q12h	PO
Leptospirosis		
Doxycycline	5 mg/kg q12h	PO
Ehrlichiosis		
Doxycycline	5 mg/kg q12h or 10 mg/kg q24h for 4 weeks	PO
Tetanus		
Metronidazole	10–15 mg/kg q8h	IV

If corticosteroid-associated adverse effects become intolerable, then adjunct treatments should be considered (12). Azathioprine at 2 mg/kg PO q24-48 h or cyclosporine at 5 mg/kg PO q12h can be combined with prednisone for several months during the tapering period (14). It is important to keep in mind the multitude of side effects and lab work changes that can occur secondary to these medications (29). Other medications that have been considered but uncommonly used in refractory cases include leflunomide at 2–3 mg/kg PO q24h and mycophenolate mofetil at 10 mg/kg PO q12h (Table 2) (28, 29). Immunosuppressive therapy is maintained until the patient regains jaw function and CK levels normalize (14). It can then be tapered slowly to lowest dose that prevents clinical sign from recurring (29). Anecdotally, many clinicians will maintain patients on immunomodulatory medications for 2–3 months following resolution before starting to taper, yet no evidence is available to suggest this is needed to prevent relapse.

A large retrospective cohort study evaluated treatment response during prednisone tapering in relation to repeated 2 M antibody titers. The study concluded that titers of 1:100 or greater indicated that prednisone needed to be increased by 25–50% once daily but still kept at an anti-inflammatory dose. Further research is needed to corroborate this method of measuring treatment response, but it could prove to be a useful monitoring tool for MMM cases that may be at risk of relapsing and redeveloping clinical signs (12).

Response rate to therapy is reported from 57.1–90.9% with conventional corticosteroid therapy (12, 19). In one study, initial clinical improvement was observed as early as three days after starting immunosuppressive therapy. By four weeks, masticatory muscle function progressively returned in 18/22 dogs. Tapering to an effective dose that kept clinical signs at bay, typically on an alternate-day schedule, started at three to four weeks after treatment initiation (12).

Relapse is an important consideration in these cases. Only one study reports on recurrence rate and found that it occurred in 27.3% of dogs from 4–66 months after initial diagnosis. Specifically, in these dogs (4/22), relapse occurred 3–60 weeks after the prednisone was discontinued. Within this cohort, the lowest reported dose that kept MMM symptoms at bay was 0.3 mg/kg once weekly in 1/22 dogs. The remainder of dogs were maintained long term at prednisone doses of 0.1 mg/kg every other day to 0.3 mg/kg q12h. Four dogs were tapered off of prednisone completely, but three of them relapsed within three months (12). In short, recovery of MMM in the acute phase is good, but some patients many require lifelong immunosuppressive therapy (14).

The use of alternative medication has also been recently described (29, 30). A small case series in three dogs examined the use of Oclacitinib, a selective janus kinase (JAK-1) inhibitor, for treatment of MMM to avoid corticosteroid adverse effects. Oclacitinib was prescribed at 1 mg/kg PO q12h as a sole therapy for MMM. The gap angle and mouth opening were increased in all three dogs in the study. Despite clinical improvement, 2 M antibody titers did not decrease. Although promising, small case numbers preclude any firm conclusions on the use of this drug in place of steroids (29).

Additionally, the use of dexamethasone was evaluated in a retrospective cohort of 17 dogs with MMM. Dexamethasone was given once at an intravenous dose of 0.2 mg/kg while in the hospital. The next day patients were discharged with oral dexamethasone at 0.1 mg/kg. Dexamethasone was the sole agent in 9/18 dogs. It was used in combination with azathioprine in 5/18 dogs and combined with cyclosporine in 2/18 dogs. One dog received a combination of all three medications. The rationale for the multidrug protocols was to help facilitate the dexamethasone taper. The researchers found that dexamethasone was non-inferior to prednisone with less adverse effects. By 10 weeks, 100% of patients showed clinical improvement (30). Once again, additional research and reports are needed to corroborate these findings.

Alternative therapies in addition to immunosuppression may also be beneficial, including therapeutic massage and acupuncture (31, 32). A single case report combined the use of prednisolone acetate with therapeutic massage. After two weeks of treatment, nearly 90% of jaw mobility was regained (31). Additionally, another single case combined the use of prednisolone acetate with acupuncture. Jaw opening gradually increased within five months of combination treatment (32). However, neither study compared the effect of combination therapy to steroids alone, nor explored the alternative therapy without the addition of corticosteroids. It is not currently possible to determine the additive effect of these interventions.

### 2.2 Polymyositis (PM)

PM is an immune-mediated and idiopathic inflammatory myopathy (9). It is classified as a type of generalized inflammatory myopathy (5). PM is not common in dogs with only 5–10 cases per million reported yearly (6). Most commonly, large breed dogs of middle age or older are affected by PM (5).

Affected dogs may develop widespread weakness and muscle atrophy that may affect the masticatory muscles (9). Clinical signs have been reported to wax and wane and can include gait stiffness, a “walking on eggshells” appearance, and lordosis (6). If laryngeal muscles are affected, then clinical signs may include dysphagia, dysphonia, and stridor (33). If esophageal inflammation is present, then clinical signs may involve megaesophagus and regurgitation (7).

Boxer and Newfoundland dogs are reported to be overrepresented in inflammatory myopathy cases (5). Both breeds have been determined to have autoantibodies targeted at muscle sarcolemma that cause breed-specific PM (5). Their IgG autoantibodies are reactive with striated muscle tissue only (6). Affected Newfoundland dogs commonly had esophageal, pharyngeal, and laryngeal dysfunction leading to dysphagia and megaesophagus (5). Several other case reports and retrospective studies further support these breed correlations with PM (5, 9, 34–36).

Additionally, a large retrospective cohort study of 369 medical records described PM in 77 Hungarian vizsla dogs characterized by regurgitation, dysphagia, and atrophy of masticatory muscles. Histologically, immune-mediated cellular infiltrates were found in the muscles of mastication along with muscles of the pharynx and esophagus (36).

The median age of diagnosis in boxer dogs is 6-years and median duration of clinical signs prior to PM diagnosis was 3-weeks (35). Interestingly, many of the cases in boxer dogs were also associated with neoplasia, specifically lymphoma and round cell tumors (34, 35).

Three potential hypotheses have been provided to link the pathogenesis of PM to lymphoma. First, T-cells responsible for disease infiltration may undergo neoplastic transformation into lymphoma. Second, extra-skeletal multicentric lymphoma is the primary disease process and PM is a secondary disease process, creating a paraneoplastic syndrome. Third, PM could be incorrectly diagnosed and patients actually have primary skeletal muscle lymphoma (PSML) (34). Multiple other case reports describe four individual cases of neoplasia-associated PM without a clear connection (37–40).

It is imperative to rule other infectious and immune causes of myopathy while working up PM cases (5). PM can present similarly to myasthenia gravis, so muscle biopsy and acetylcholine receptor antibody testing can be done to differentiate the two diseases (6).

A few key serum chemistry changes have been identified in dogs with PM (9). A large, retrospective, clinicopathologic review of 200 cases found that CK and AST values were significantly higher for generalized inflammatory myopathies, including PM, than focal inflammatory myopathies (5). A consistently elevated CK value on chemistry is indicative of ongoing myofiber necrosis and supports further workup with a muscle biopsy (34). Histologically, PM is characterized as a myopathy that contains mononuclear cell infiltrates without a true underlying disease process (5). Although the target antigen on muscle cells has not been identified, effector cells include T cells (helper and cytotoxic), macrophages, and natural killer cells (34). Thus, there is infiltration of immune cells within affected muscles in PM (9). This makes PM both immune-mediated and idiopathic in nature (7).

MRI is the most commonly used advanced imaging modality for PM cases in humans (8, 9). Characteristic findings include hypointense T1 weighted images and hyperintense T2 weighted images (8). After contrast administration, there is enhancement that is distinct and uneven. Visualization of inflammatory changes can be enhanced with use of STIR sequences (9).

The mainstays of treatment are similar to other inflammatory myopathies and commonly include prednisone, prednisolone, and azathioprine at immunosuppressive levels (9, 36). The retrospective cohort study on Hungarian vizsla dogs reported combination therapy of prednisolone and azathioprine comprised 86% of reviewed cases. A corticosteroid combined with cyclosporin, leflunomide, or methotrexate was utilized in the remaining 14% of cases. Initial dosages utilized in the study included the following: glucocorticoids at 1–2 mg/kg PO q12h, azathioprine at 2 mg/kg PO q24h, leflunomide at 4 mg/kg PO q24h, and methotrexate at 2.5 mg/m^2^ PO q24h (Table 2). Individual treatment response rates were not listed in this study. Instead, it was reported that 90% of all dogs showed improvement in clinical signs.

## 3 Generalized myositis of infectious origin

Infectious causes of generalized myositis can be parasitic, bacterial, rickettsial, or fungal in origin (7). An important differentiating factor for dogs affected with infectious myositis is that they are more likely to display lower motor neuron weakness (5). In a review of 200 cases of inflammatory myopathies, 140 cases were determined to be generalized inflammatory myopathies and 28.5% of those were classified as infectious (5). A presumptive diagnosis of generalized myositis of infectious origin may be based on clinical signs and positive serology testing, but a definitive diagnosis requires organism identification through immunohistochemical or molecular means (7).

Parasitic etiologies are most commonly reported and include neosporosis, toxoplasmosis, leishmaniasis, hepatozoonosis, trypanosomiasis, trichinosis, sarcocystosis, and microfilariasis (6, 7). Of these, *Neospora caninum* is the most common parasitic cause of infectious myositis (6). Transmission occurs through ingesting tissues containing bradyzoites such as fetal membranes or placentas. Dogs can also be infected vertically through placental transmission of tachyzoites (41). Clinical signs of dogs with neosporosis include stiffness, muscle atrophy including the masticatory muscles, and myalgia. A vast majority of dogs will also exhibit polyradiculoneuritis and display characteristic pelvic limb hyperextension and rigidity. *N. caninum* may lead to atrophy through secondary denervation or radiculoneuropathy (6). MRI imaging of dogs affected with *N. caninum* shows bilateral and multifocal changes to masticatory muscles on T2 weighted and fluid-attenuated inversion recovery (FLAIR) images. Contrast administration causes enhancement of muscular lesions (42). Definitive diagnosis requires use of tissue aspirates or muscle biopsies to detect tachyzoites and bradyzoites, respectively. Although there is no approved treatment protocol for *N. caninum*, the following medications have been used for clinical control: clindamycin at 12–25 mg/kg PO q12h for 4 weeks or trimethoprim sulfadiazine 15–20 mg/kg PO q12h for 4 weeks with pyrimethamine at 1 mg/kg PO q24h for 4 weeks (Table 2) (41).

*Toxoplasma gondii* infections are highest in populations of dogs that consume raw or undercooked meat containing bradyzoites (7). Additionally, many cases of *T. gondii* infections have been reported in young, immunocompromised dogs with concurrent canine distemper viral infections (43, 44). Clinical signs are related to central nervous system dysfunction, but myopathic changes include myalgia, atrophy, stiffness, and inflammation (45). Similar to *N. caninum*, polyradiculoneuritis may also be present with hyperextension and rigidity of the pelvic limbs (46). Affected muscles may develop granulomatous inflammatory reactions that correspond to bradyzoites (7). Diagnosis may be performed through antibody assays and PCR tests through use of feces, fluid, or tissue (47). Successful treatment options that have been reported in the literature include clindamycin at 10 mg/kg PO q8h or trimethoprim sulfadiazine at 15 mg/kg PO q12h with pyrimethamine at 1 mg/kg PO q24h (46). Additionally, azithromycin at 10 mg/kg PO q24h has been used in cases of disseminated disease (Table 2) (47).

Infection with *Leishmania* has also been reported to cause masticatory and skeletal myositis in dogs (6, 7). A retrospective cohort study of 24 dogs diagnosed with leishmaniasis found a commonality of bilateral masticatory muscle atrophy and myalgia, yet normal jaw function (48). Additional clinical signs of systemic leishmaniasis include lymphadenopathy, weight loss, severe dermatitis, and weakness (7, 43). Dogs affected with leishmaniasis show myopathic changes including necrosis, fibrosis, and mononuclear cell infiltration (lymphocytes, histiocytes, and plasma cells) on histopathology reports (43). For a definitive diagnosis, identification of Giemsa-stained amastigotes is needed (48). The amastigotes are found within macrophages at site of infection and not within skeletal muscle fibers (49). There is a positive correlation between the quantity of amastigotes present and severity of disease and inflammatory change (43). The “classic” treatment option for canine leishmaniasis is a pentavalent antimonial, specifically meglumine antimoniate (Glucantime) (49). Meglumine antimoniate is dosed at 75–100 mg/kg SQ q24h for 4–8 weeks and allopurinol is dosed at 10 mg/kg PO a12h for at least six months (50). A second-choice treatment option is amphotericin B dosed at 0.1–1 mg/kg IV q24h or twice weekly for up to two months. More recently, the combination of miltefosine dosed at 2 mg/kg PO q24h with allopurinol dosed at 10 mg/kg PO q12h for six months has been described (Table 2) (51).

Trypanosomiasis (Chagas disease) caused by *Trypanosoma cruzi* affects skeletal and myocardial muscles in dogs. Disease progression can be acute or chronic (7). Acute manifestations of *Trypanosomiasis* can lead to rapid death due to myocarditis and arrhythmias. Chronic manifestations lead to severe cardiac changes such as dilated cardiomyopathy and eventually results in congestive heart failure (52). No current cases have been reported to affect canine masticatory muscles, but the potential is there. Definitive diagnosis requires the identification of trypomastigotes within a Wright’s or Giemsa-stained blood smear (7). Treatment can be challenging and complex in cases of Chagas disease. The use of congestive heart failure medications with amiodarone at 7.5 mg/kg PO q24h and itraconazole at 10 mg/kg PO q24h has been trialed (Table 2) (53, 54).

Canine sarcocystosis is rare, but it has been documented in a handful of case reports to cause generalized myositis (55, 56). A case report of two dogs in the United States reported clinical signs of fever, myalgia, muscle atrophy, and reluctance to move. Clinicopathology changes included increased ALT, lymphopenia, and thrombocytopenia. Muscle biopsies were taken and histologic examination revealed the presence of many sarcocysts and a necrotizing and inflammatory myopathy. Analgesics and clindamycin at 13 mg/kg PO q12h were initially prescribed for both patients; one dog eventually died and the other responded with the addition of decoquinate at 10–20 mg/kg PO q12h (Table 2) (55). Additional research is needed on the efficacy of decoquinate in canine sarcocystosis.

Bacterial diseases are much less common causes of infectious myositis, yet include leptospirosis and clostridial infections. Some cases of *Leptospira australis* and *Leptospira icterohemorrhagiae* infections have resulted in generalized myositis. Clinical signs observed in these cases are severe myalgia, fever, and difficulty walking (7). Supportive laboratory diagnostics include a *Leptospira* microscopic agglutination test (MAT) titer ≥ 800 or presence of IgM antibodies. Definitive laboratory diagnostics include at least a fourfold increase in the *Leptospira* agglutination titers from acute phase and convalescent phase samples. Treatment includes a two-week regimen of doxycycline at 5 mg/kg PO q12h (Table 2) (57).

Clostridial infections in myositis cases have been reported to cause severe myalgia (7). Infection introduction has been documented from penetrating wounds, intramuscular injection sites, and surgical contamination (57–59). Aggressive debridement and strain-specific antibiotic therapy is required in these cases (7). Infection specifically with *Clostridium tetani* will be discussed in more detail in the following section.

A rickettsial disease that has been reported to cause infectious myositis includes *Ehrlichia canis*, causing canine monocyte ehrlichiosis (7). Early on in ehrlichiosis, dogs have been reported with PM due the presence of muscle atrophy and cellular infiltrates being identified in muscle biopsies (6). Affected dogs can go through acute, subclinical, and chronic phases of disease (60). The acute phase is when dogs have been documented to develop myositis and clinical signs such as diffuse atrophy (7). Thrombocytopenia and anemia occur and cause pale mucous membranes to be present (60). Morula of *E. canis* are detected only 4–6% of the time, so additional testing through serology and PCR are required for diagnosis (61). Treatment consists of tetracycline antibiotics such as doxycycline at 5 mg/kg PO q12h or 10 mg/kg PO q24h for four weeks (Table 2) (62). Tick-borne diseases may also cause infectious myositis with similar clinical signs to *N. caninum,* including *Hepatozoon canis and Hepatozoon americanum* (6). Muscles biopsies of those with an infectious myopathy contain characteristic parasitic cysts (5).

In short, more than one test is typically required to diagnose an inflammatory myopathy and determine its etiology. This can range from any combination of serologic testing for infectious agents or autoantibodies, clinical signs, blood work, or muscle biopsy with immunophenotyping. MMM and PM can be differentiated by use of 2 M antibody titer testing and muscle biopsy. There is potential for diagnostic results to support both MMM and PM, in which OS may be present. Histologically, dogs with MMM typically have perimysial and endomysial fibrosis and infiltrates while dogs with PM have only endomysial fibrosis and infiltrates. Parasitic cysts can be identified in biopsies of cases with infectious myositis. Clearly, biopsy is key in the diagnostic workup of inflammatory myopathy cases (5).

## 4 Trauma

Trauma affecting the muscles of mastication may result in contracted or swollen masticatory muscles further resulting in restrictive mouth opening (RMO). Other traumatic injuries can occur to the bones of the skull near the muscles of mastication and cause trismus as a secondary process.

Another potential consequence of masticatory muscle trauma is acute compartment syndrome (ACS). This is a rare musculoskeletal condition in which there is an acute increase in osteofascial compartment pressure (63). Multiple authors suggest that ACS develops when the pressure within the affected area nears systemic diastolic blood pressure (64, 65). If left untreated, it may cause ischemia and necrosis of the affected area (65), carrying a high morbidity rate (64).

Diagnosis of ACS can be challenging and is done on an emergent basis (65). Clinical diagnosis is dependent on the combination of clinical signs and measurements of intra-compartment pressures (64). Previous cases in veterinary literature have reported that pressures exceeding 30 mmHg require immediate fasciotomy (63). CT imaging of ACS in dogs reveals nonhomogeneous contrast enhancement and enlargement of affected muscles (66). Treatment for ACS involves emergency fasciotomy for surgical decompression (66). A review of ACS in human medicine indicated that the best prognosis occurs if surgery takes place within six hours of the inciting injury or traumatic event. After six hours, the chance of permanent nerve injury is increased. If necrotic tissue is present, debridement is necessary and there is an increased chance of infection (65).

**Figure 3:**
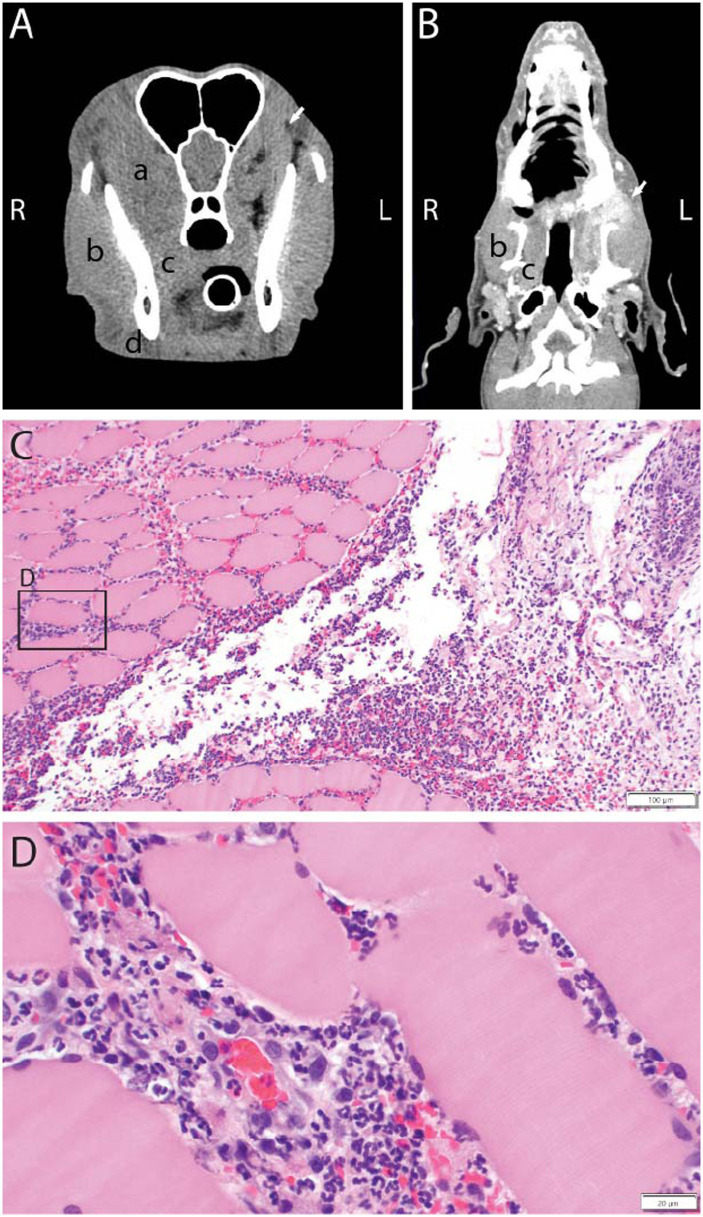
Transverse **(A)** and dorsal plane reconstruction **(B)** contrast CT of a 1-year-old female spayed golden retriever that was presented for pain on opening the mouth and facial swelling on the left side of the face. Noted on the unaffected side are the temporalis muscles (a), masseter (b), pterygoid (c), and digastricus (d). The dog was diagnosed with left retrobulbar inflammation and potential abscess with extension of the inflammation to the left temporalis muscle likely due to penetrating trauma with inoculation or unidentified foreign material. Biopsy and histology of the left temporalis muscle revealed cellulitis and myositis are primarily neutrophilic which is suggestive of a bacterial infection and culture and sensitivity testing of the biopsy revealed *Fusobacterium nucleatum* (beta-lactamase negative) bacteria (**C** x10 magnification, **D** x40 magnification H&E).

Penetrating foreign bodies may also affect the muscles of mastication (Figure 3). A report on two dogs with oropharyngeal foreign bodies localized to the pterygoid muscle were diagnosed via CT. Both dogs had chronic histories (one year and five years, respectively) of discomfort and inability to fully open the mouth. CT imaging revealed asymmetric and contrast-enhanced regions of the pterygoid muscle (67). Similarly, another report documented an 8-year-old German shepherd with trismus and mouth pain for one month duration along with fever and temporalis muscle atrophy. MRI findings were supportive of myositis of both temporalis muscles, and a subcutaneous abscess was detected dorsal to the sagittal crest. It was suspected that the abscess was due to a penetrating injury from chewing on a stick (68).

Infection and abscess secondary to a stick chewing penetrating wound is typically treated with antibiotics such as amoxicillin-clavulanic acid (68). Alternatively, surgery may be pursued in trauma cases as needed if a foreign body is identified in the masticatory muscles or its proximity (67–72). Removal of pterygoid muscle foreign bodies and necrotic material was facilitated through a novel paramedian ventral mandibular approach in the report on two dogs (69).

Post trauma physical therapy to the craniofacial region is not yet well established in veterinary medicine. The main goal of physical therapy is to restore and maintain adequate range of motion and decrease fibrotic changes. Physical therapy sessions may involve massaging the affected muscles and then opening the mouth and holding that position for 20–30 s; the hold is then released and the exercise may be repeated (69). The authors have also discussed using OraStrech (CranioMandibular Rehab, Inc. Wheat Ridge, Colorado) to rehabilitate the jaws and masticatory muscles in various circumstances, but studies demonstrating its efficacy in dogs are currently unavailable (1). Also, implementing diet changes such as progressively larger kibble to encourage chewing can be utilized in the post-operative period (69).

## 5 Myositis ossificans (MO)

MO is defined as non-malignant, heterotopic, metaplastic bone that develops within skeletal muscle (72). MO cases typically present with inability to open or close the mouth depending on the location and extent of mineralization. MO is subdivided into myositis ossificans progressiva (MOP) and myositis ossificans traumatica (MOT), which are differentiated by their pathophysiology and etiology (73, 74).

MOP is a rare hereditary disorder with a human prevalence of 1/2,000,000 reported (75). Historically, it has been described by several other names including Münchmeyer’s disease and “stone man syndrome” in humans (76). More recently, MOP has been referred to as fibrodysplasia ossificans progressiva (FOP) in all species (74).

The human inheritance pattern has been determined to be autosomal dominant and caused by a mutation in the Activin A receptor type 1 (*ACVR1*) gene, which encodes for Activin receptor-like kinase 2 (*ALK2*) receptor for bone morphogenic proteins (BMP) (75–77). Mutation of this gene in MOP patients results in dysregulation of BMP pathways (78), and *ALK2* is involved in signaling pathways that regulate the formation of new bone, fracture healing, and embryology (76). Since the inhibitory protein for *ACVR1/ALK2* is partially deleted in this mutation, *ACVR1/ALK2* is active in the absence of BMP signaling (77). This leads to an overabundance of osteoblastic activity at the site of overexpression and development of MOP lesions that can be multifocal (76). The genetic basis of canine MOP has not been established and requires further research.

In humans, initial masticatory muscle involvement is not common in MOP but can occur with disease progression (76). A review of 42 human cases of MOP affecting the masticatory muscles found that the masseter is most commonly affected. This is followed by the medial pterygoid, lateral pterygoid, and temporalis in order of decreasing frequency of occurrence (79).

Cases in the literature of MOP or MOP-like lesions in dogs are rare and typically involve lameness and target the hip joint (80, 81). There are two individual case reports of canine MOP lesions developing within paravertebral muscles near the cervical spine (82, 83). One report described a neurogenic lameness of the left forelimb, which was the first reported case of canine MOP causing nerve compression (82). The other report did not describe any neurologic deficits (83). Currently, no cases have been described of MOP lesions within canine masticatory muscles.

MOT is a localized, idiopathic, heterotopic osseous lesion formed within soft tissue structures (84). It has also been described by several other names including traumatic myositis ossificans, myositis ossificans circumscripta, localized myositis ossificans, or fibrodysplasia ossificans circumscripta (85). Although no etiology has been agreed upon in human or veterinary medicine, some suspect MOT occurs in response to a traumatic injury that creates fibroproliferative change, forming mature endochondral bone (86). Further, it is hypothesized that BMPs modulate this response and are directly released from injured bones secondary to trauma (85). Other theories involve the notion of bony fragments translocating into nearby soft tissues following trauma, movement of subperiosteal osteoprogenitor cells, hematoma formation with secondary mineralization, or periosteal detachment and osteoprogenitor cell proliferation (87).

In human literature, proposed inciting injuries of MOT affecting the muscles of mastication include external traumas, tooth extraction complications, anesthetic injections, odontogenic infections, and burns (73, 88). In dogs, there is a single maxillofacial case report of MOT occurring in a 5-month-old female intact German shepherd after experiencing a dog bite wound below her right zygomatic arch (89). A majority of reported cases of canine MOT have been described in the triceps muscle, caudal thigh muscles, caudal cervical region, and extensor carpi radialis muscle following traumatic or concussive injuries (83, 90–92).

There is a suspected correlation between MOT and hemophilia in humans, specifically with Factor XI deficiency (80). Similarly, there is a proposed link between Doberman pinschers and the development of MOT-like lesions in the face of microvascular bleeding (80).

MO of the pterygoid muscles has been described in dogs and humans from both genetic and traumatic etiologies (1, 88, 93). If a genetic basis is not found, human causes of pterygoid mineralization are typically from traumatic events ranging from dental extractions to vehicular accidents (88). A small case series described two young French bulldogs with bilateral MO-like lesions within their pterygoid muscles, causing trismus. The history of both patients was unknown, so it was unclear if these were cases of MOP or MOT within the pterygoids (1). Another case report identified similar bilateral ossification in the area of the pterygoid muscles in an Airedale terrier, but it was not called MO. Instead, it was referred to as extracapsular soft tissue ossification (93).

**Figure 4:**
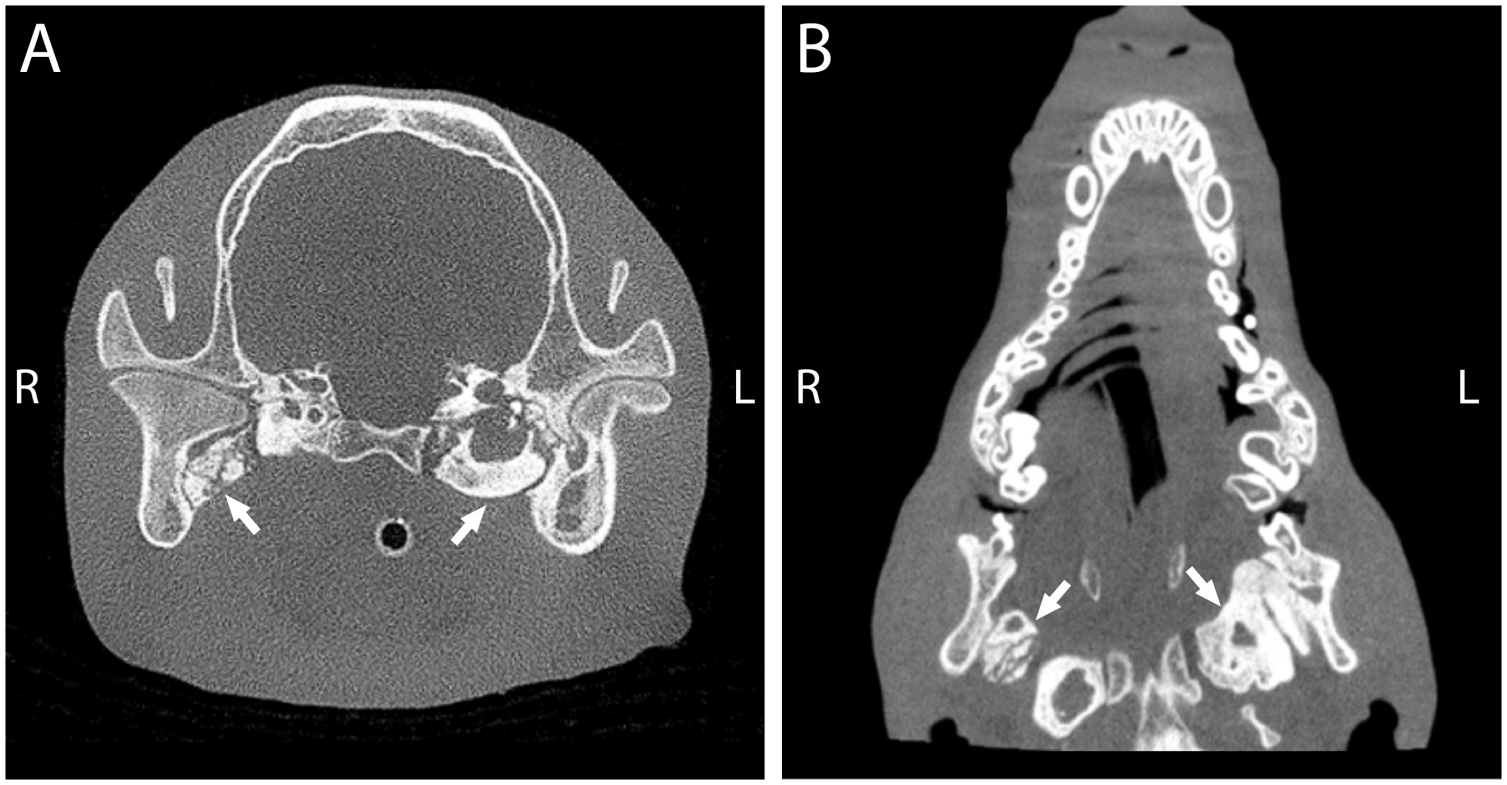
Transverse **(A)** and dorsal plane reconstruction **(B)** cone-beam computed tomography of a 1-year-old mixed breed dog with inability to open the mouth. Imaging demonstrated bilateral ossification of the pterygoid muscles including osseous proliferation that bridges from the medial aspect of the caudal mandible to the tympanic bulla (arrows).

The diagnostic workup for suspected MO cases includes blood work, advanced diagnostic imaging, and biopsy with histopathology (85). Bloodwork can rule out other systemic conditions that cause extra-skeletal ossification and should include assessment of phosphatase, parathyroid hormone, and calcitonin levels (87). Diagnostic imaging should include MRI or CT to fully assess maxillofacial structures and for early disease detection (73). When using MRI, both T1 and T2 weighted images of mature MO lesions appear hyperintense and similar to cancellous fat within affected muscles (82). On CT, MO appears as an ossified growth that has a zonal pattern with increasing radiopacity centripetally and a radiolucent center (73, 87). In pterygoid MO cases, common CT findings include osseous proliferation that bridges from the medial aspect of the caudal mandible to the tympanic bulla (Figure 4) (94).

**Figure 5:**
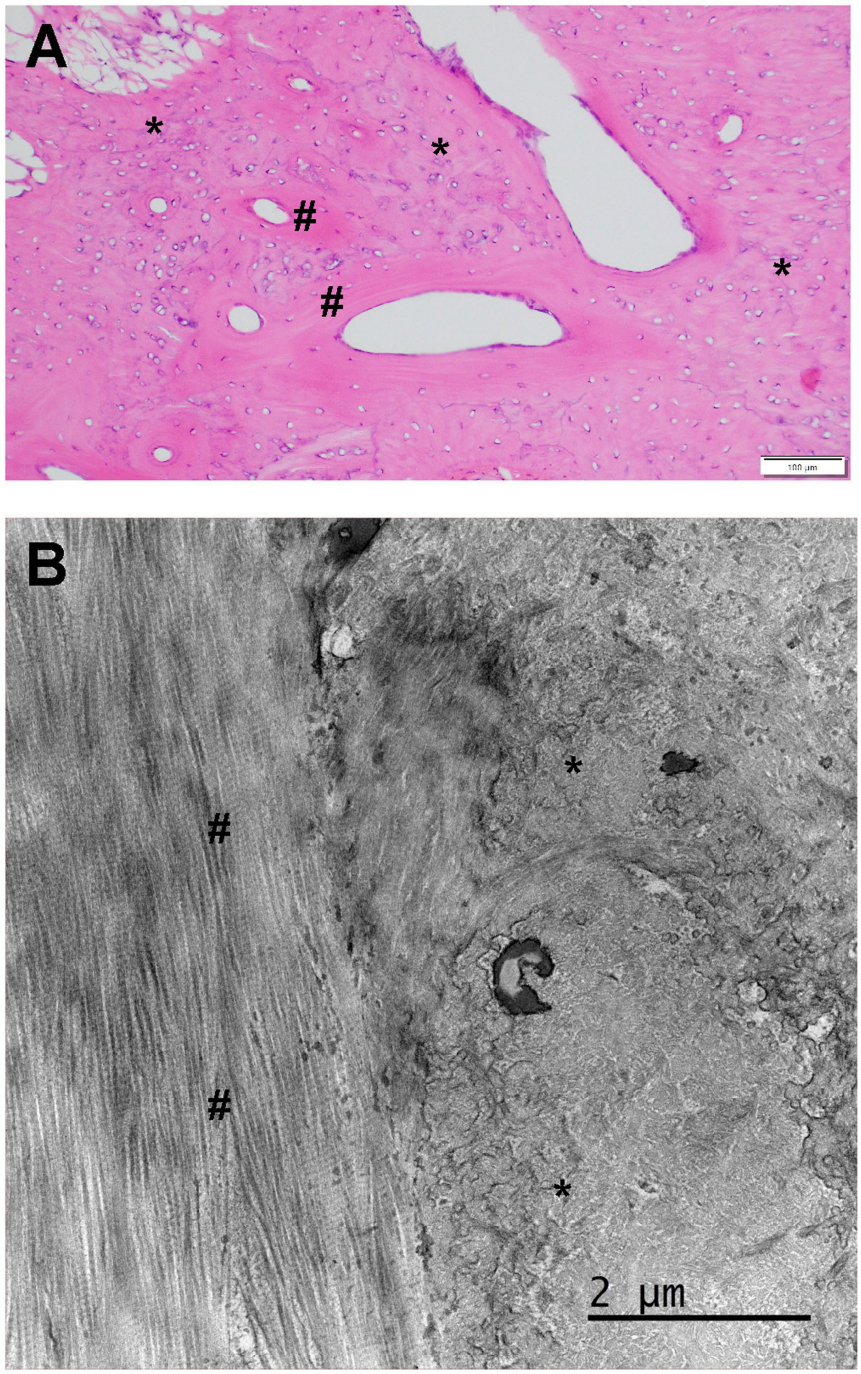
**(A)** Standard hematoxylin and eosin (HE) histology obtained from a muscle biopsy from a 7-month-old French bulldog exhibiting bilateral ossification of the pterygoid muscles. There was no apparent muscular tissue in the section presented. However, there was a mixture of mature lamellar (number sign) and immature woven bone (asterisk). The clear oval spaces were probably occupied by blood vessels that were lost in the processing. **(B)** Transmission electron microscopy for the interface between the woven (right, asterisk) and lamellar bone (left, number sign). Note the regimented and parallel collagen fiber alignment in the lamellar bone and the more haphazard and scrambled appearance of the collagen fibers in the woven bone. The dark areas represent mineralized portions of the bone matrix that were not completely removed by the decalcification process. From De Paolo et al. (1).

Although imaging is typically suggestive of MO, definitive diagnosis is made through biopsy and histopathology (95). Histological changes consistent with MO have been described as a “zone phenomena,” meaning that there are three identifiable zones (inner, middle, and outer) (85, 94). The inner zone houses undifferentiated cells along with muscular tissue that can contain hemorrhagic and necrotic components. Mitosis can be detected in giant mesenchymal cells present in the inner layer. The middle zone includes immature osteoid and active osteoblasts. Woven bone tissue is also detected in the middle zone. Finally, the outer zone is where mature lamellar bone can be found with evidence of osteoclastic activity (85). Collectively, these findings indicate that osseous metaplasia or heterotopic mineralization has taken place (Figure 5) (94). In short, the clinical diagnosis of MO requires histopathologic detection of the progression from fibrous metaplasia to mature bone in affected muscles (1).

Clinically, it is important to distinguish MOP from MOT due to MOP having a worse prognosis (76). MOP cases are typically multifocal, so surveying other areas of the body for MOP lesions is recommended. Ultimately, MOP is a progressive and disabling disorder that typically leads to ankylosis and immobilization of affected soft tissue structures (75). Disease prognosis is poor with no current successful treatment options (76). Death is usually secondary to thoracic insufficiency syndrome (75).

Conversely, MOT is typically localized to a single area of the body and patients may have a history of traumatic injury (73, 94). Additionally, follow up with serial imaging, such as CT, may show that MOT lesions remain static with lack of progression (1).

Treatment options range from conservative management to surgical excision of ossified lesions (85). There is no definitive medical management for MO in humans or animals, and current medical management is aimed at reducing pain, optimizing function, and avoiding additional trauma (95). In humans, MO lesions and flare ups localized to the back or chest can be managed with cyclooxygenase-2 (COX-2) inhibitors, non-steroidal anti-inflammatories (NSAIDs), or muscle relaxants; MO lesions and flare ups of the limbs and jaw in humans can be managed with prednisone and muscle relaxants. Use of leukotriene inhibitors, mast cell stabilizers, and bisphosphonates have also been explored in human medicine but require more studies on their efficacy (95). Physiotherapy may be implemented in cases of conservative management (85). Future research is needed specifically for MOP treatment to target BMP signaling pathways mediated by *ACVR1*.

Thoughtful surgical planning is required for MO cases and timing of surgical intervention is controversial (1). It is believed that if surgery is performed while lesions are still developing during the acute or immature phase, then it could lead to more aggressive bone development and recurrence (74, 85). The acute phase of MO development can last two to six weeks and is followed by the mature phase in which complete ossification occurs over several months (96). Therefore, many authors support surgical intervention during the mature phase once lesion ossification is present; this allows for the lesion to be well delineated from skeletal muscles surrounding it (85). In severe cases, such as bilateral pterygoid mineralization, salvage procedures such as bilateral segmental mandibulectomy may be appropriate (1).

Recent research in human medicine has explored the use of peri- and post-operative radiation therapy in surgical management of MO cases and helping decrease recurrence rates. The current radiation therapy recommendation to decrease MO recurrence in humans is 7 to 8 Gray (Gy) in a single fraction 24 h before surgery or 48 h after surgery (97). If surgical intervention is not possible and conservative therapy is not effective, then euthanasia may need to be discussed. This is evident given poor patient welfare and potential for life threatening complications in severe cases of MO affecting the maxillofacial region and causing inability to open the mouth (1).

## 6 Tetanus

Tetanus is a neurologic disease caused by *Clostridium tetani,* a sporulating, anaerobic bacterium found in soils and characterized by a spastic paralysis (98, 99). *C. tetani* produces a neurotoxin termed the tetanus toxin (TeNT) along with a hemolysin known as tetanolysin (100). Tetanus infection may present as a localized or systemic form (101). Systemic (generalized) cases of tetanus start off with spasticity of the masticatory muscles, creating a “sarcastic grin” (also known as *Risus sardonicus*) appearance, ‘locking of the jaws’ and progressing to involvement of the trunk and extremity muscles (99).

The most commonly affected areas of the body are those that make contact directly with soil (100). Wound conditions that favor spore germination are anaerobic with necrotic tissue present and little access to air (101). Once a wound is contaminated with *C. tetani*, tetanus takes place when its toxin is released (100). The authors have also seen a fractured deciduous canine tooth that was the most likely nidus of infection (unpublished data).

At the site of infection, TeNT is exposed to local demyelinated nerve endings. Once TeNT is bound to its receptor (currently not known) it is transported to the central nervous system (CNS) to a neuronal cell body via retrograde axonal transport (102). Its final destination is inhibitory interneurons in the CNS that are responsible for coordinating motor nerve activity. TeNT then inhibits the release of gamma-aminobutyric acid (GABA) and glycine (103). When both of these molecules are inhibited, it leads to continued muscle contraction due to excessive motor neuron firing (100). Tetanospasmin also inhibits additional neurotransmitter release including acetylcholine (102).

A retrospective analysis of 18 cases of tetanus in dogs described that the first clinical signs involve localized maxillofacial changes such as trismus, dysphagia, vomiting, and *risus sardonicus*. Nearly 50% of infected dogs progress to display signs of systemic (generalized) tetanus. Clinical signs of progression involve muscle contractions that are tonic and prolonged in nature (104). Since voluntary muscles are targeted, clinically affected animals present with tetanic spasm and rigidity (101). Affected animals may also experience autonomic nervous system changes in which sweating, hypertension, and tachycardia develop and are followed by hypotension and bradycardia (100). The duration of clinical signs may be several weeks (101).

A retrospective cohort study of 53 cases of canine tetanus reported respiratory complications in 26.4% (14/53) of patients, including aspiration pneumonia and upper airway obstruction. A poor prognosis was associated with these patients and only 14.3% (2/14) survived. This was contrasted to dogs without respiratory complications in which 94.8% (37/39) survived to discharge (98).

Diagnosis of tetanus is initially presumed based on the presence of spastic paralysis +/− presence of a wound, as there is no current definitive antemortem lab test for tetanus (104). Early cases of clinical tetanus may be misdiagnosed as myopathies. In dogs, localized tetanus may also be diagnosed with use of EMG (101). Doublets and triplets will be recorded and are consistent with motoneurons that are hyperexcitable. Repeatability of EMG findings under general anesthesia is ideal for confirmation of motor neuron disease (102). If present, wound exudate may be used to isolate *C. tetani* for culture. *C. tetani* may lose its ability to pick up Gram coloration in Gram staining tests if the culture is greater than 24 h old. Finally, advanced diagnostic imaging in the form of MRI or CT typically reveals inflammatory change (102).

There is no specific treatment for active tetanus infections other than supportive care (100). Analysis of survival in a retrospective study of 42 cases found that young dogs (less than two years of age) with severe generalized disease and rapid progression have a guarded prognosis (99). Mortality ranges from 18 to 50% with higher rates in dogs with respiratory and autonomic complications (101).

Wound cleaning, debridement, and antibiotic therapy such as metronidazole at 10–15 mg/kg IV q8h are a priority in tetanus cases (Table 2) (100, 105). Tetanus antibodies can be utilized to stop unbound TeNT from continued binding, but it cannot prevent the action of TeNT that is already in the CNS (100). It is imperative to make sure patients are hospitalized in a location that is quiet, dark, well ventilated, and has minimal stimulation to prevent spasms (101). A few clinical case reports have used magnesium sulphate (MgSO_4_) as an adjunct muscle relaxant in cases of canine generalized tetanus (106, 107).

## 7 Neoplasia

Neoplasia is a differential that should also be considered in cases of masticatory muscle dysfunction. Neoplasms may restrict mouth opening directly by involving the muscles of mastication or indirectly by involving the temporomandibular joint, trigeminal nerve or its branches, other soft and osseous tissues, or by causing cancer cachexia (108).

Additionally, neoplasia typically leads to unilateral masticatory muscle atrophy and facial asymmetry. A neoplastic process may be localized to the trigeminal nerve such as a peripheral nerve sheath tumor (109). Other less common types of neoplasia that may affect the trigeminal nerve include lymphoma (4). Besides unilateral atrophy, affected dogs may also have neurologic deficits such as absent palpebral reflex, decreased menace response, loss of corneal sensation, ipsilateral Horner’s syndrome, and enophthalmia (109).

A retrospective cohort study of 64 cases of dogs with unilateral masticatory muscle atrophy reported that MRI was unable to localize a lesion, such as a tumor, in over 25% of cases. Preliminary discussion on this topic proposed that the lesions may be present but are too small to be detected on MRI. Additional studies are needed to support this notion and/or identify the pathology present in that group lacking a detectable discrete tumor (109).

## 8 Conclusion

This comprehensive review included multiple areas further research related to the diagnosis and treatment of masticatory muscle disorders in dogs. Diagnostic uncertainties include the limitation of MRI for detection of neoplasia. Additionally, the presence of an OS with characteristic findings of both MMM and PM creates another level of diagnostic uncertainty in these cases. Therapeutic uncertainties revolve around MMM and establishing a universally accepted protocol for starting immunosuppressive dosages of corticosteroids, tapering timeline, monitoring, and long-term management of refractory cases. The alternative use of dexamethasone, azathioprine, mycophenolate mofetil, leflunomide, cyclosporin, and JAK inhibitors for treatment of MMM also requires further research to establish efficacy. Finally, additional investigation is needed to determine the genetic basis of MOP in dogs along with the potential for targeted treatment at BMP signaling pathways.

**Figure 6:**
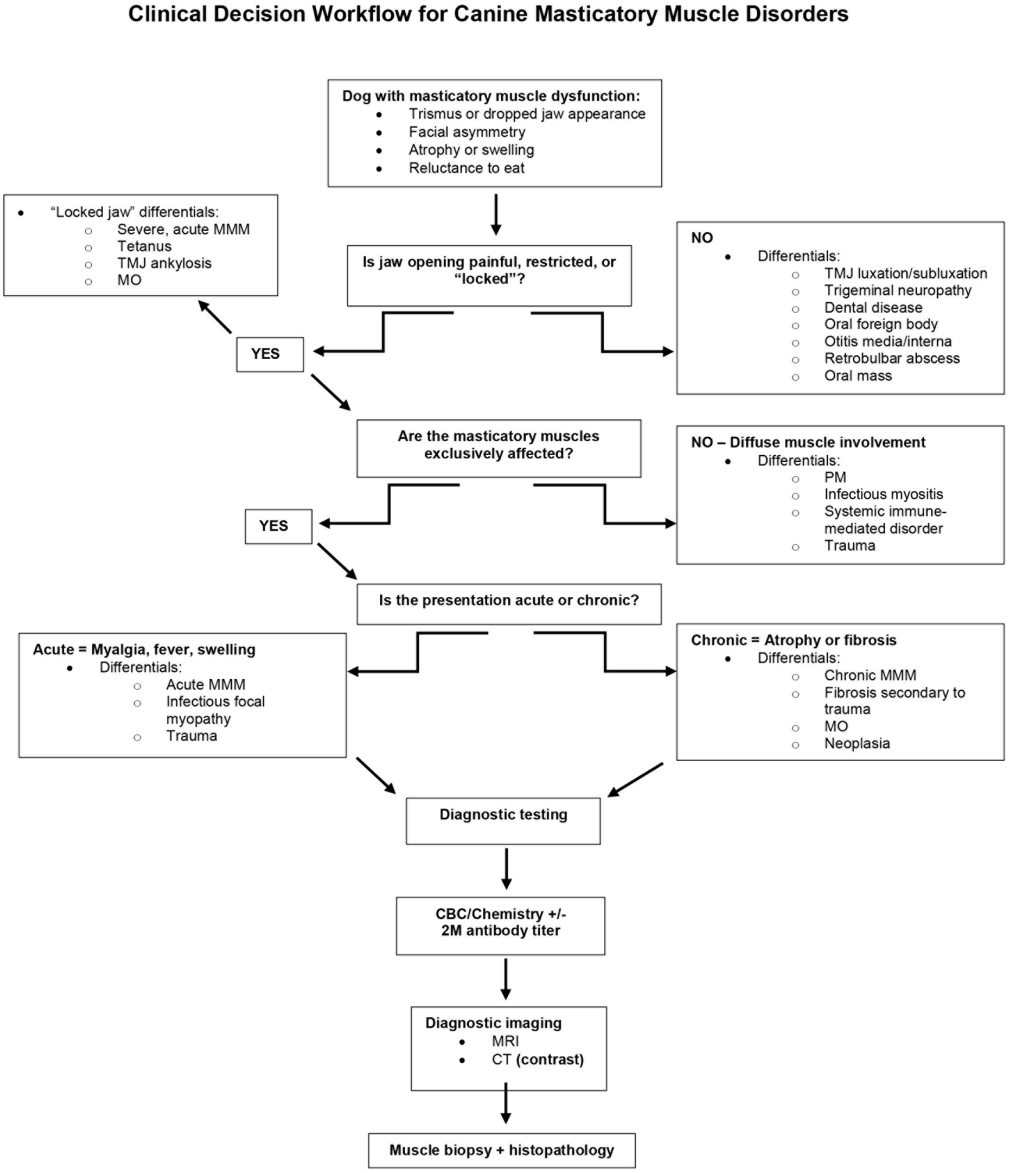
Decisional algorithm for workup and diagnosis of disorders affecting the masticatory muscles in the dog.

Without a doubt, several differentials should to be thoughtfully considered in cases involving masticatory muscle dysfunction. Long term prognosis improves with early recognition, appropriate diagnostics, and targeted treatment. A working knowledge of the pathophysiology and etiology of these disorders allows for clinicians to more confidently approach cases involving masticatory malfunction. Additionally, rule out lists are fundamental in the diagnostic process of patients with a change in their mouth opening or closing. As previously established, empirically treating these patients with immunosuppressive medications can have devastating repercussions in the face of an undiagnosed infection. In short, thorough workup using advanced diagnostic imaging, biopsy, and targeted informed treatment are imperative for patients with masticatory muscle pathology due to potentially life-threatening consequences (Figure 6).

## Glossary

CN V: Trigeminal nerve
CN VII: Facial nerve
MMM: Masticatory muscle myositis
PM: Polymyositis
EOM: Extraocular myositis
DM: Dermatomyositis
OS: Overlap syndrome
CK: Creatine kinase
ALT: Alanine aminotransferase
AST: Aspartate aminotransferase
CT: Computed tomography
MRI: Magnetic resonance imaging
mMyBP-C: Masticatory myosin binding protein-C
CKCS: Cavalier King Charles spaniel
GE STIR: Gradient-echo short T1 inversion recovery
EMG: Electromyography
JAK-1: Janus kinase 1
PSML: Primary skeletal muscle lymphoma
FLAIR: Fluid-attenuated inversion recovery
RMO: Restrictive mouth opening
ACS: Acute compartment syndrome
MO: Myositis ossificans
MOP: Myositis ossificans progressiva
MOT: Myositis ossificans traumatica
FOP: Fibrodysplasia ossificans progressiva
*ACVR1*: Activin A receptor type 1
*ALK2*: Activin receptor-like kinase 2
BMP: Bone morphogenic protein
COX-2: Cyclooxygenase-2
NSAIDs: Non-steroidal anti-inflammatories
Gy: Gray
TeNT: Tetanus toxin
CNS: Central nervous system
GABA: Gamma-aminobutyric acid
MgSO_4_: Magnesium sulphate
CMO: Craniomandibular osteopathy
SLE: Systemic lupus erythematous
OA: Osteoarthritis
TNST: Trigeminal nerval sheath tumor
